# Anthocyanins from *Rubus fruticosus* L. and *Morus nigra* L. Applied as Food Colorants: A Natural Alternative

**DOI:** 10.3390/plants10061181

**Published:** 2021-06-10

**Authors:** Erika N. Vega, Adriana K. Molina, Carla Pereira, Maria Inês Dias, Sandrina A. Heleno, Paula Rodrigues, Isabel P. Fernandes, Maria Filomena Barreiro, Dejan Stojković, Marina Soković, Márcio Carocho, João C. M. Barreira, Isabel C. F. R. Ferreira, Lillian Barros

**Affiliations:** 1 Centro de Investigação de Montanha (CIMO), Instituto Politécnico de Bragança, Campus de Santa Apolónia, 5300-253 Bragança, Portugal; erimavega@gmail.com (E.N.V.); akmolinavg@gmail.com (A.K.M.); maria.ines@ipb.pt (M.I.D.); sheleno@ipb.pt (S.A.H.); prodrigues@ipb.pt (P.R.); ipmf@ipb.pt (I.P.F.); barreiro@ipb.pt (M.F.B.); mcarocho@ipb.pt (M.C.); jbarreira@ipb.pt (J.C.M.B.); iferreira@ipb.pt (I.C.F.R.F.); 2 Laboratory of Separation and Reaction Engineering-Laboratory of Catalysis and Materials (LSRE-LCM), Polytechnic Institute of Bragança, Campus Santa Apolónia 1134, 5301-857 Bragança, Portugal; 3 Department of Plant Physiology, Institute for Biological Research “Siniša Stanković”-National Institute of Republic of Serbia, University of Belgrade, Bulevar Despota Stefana 142, 11000 Belgrade, Serbia; dejanbio@ibiss.bg.ac.rs (D.S.); mris@ibiss.bg.ac.rs (M.S.)

**Keywords:** *Morus nigra* L., *Rubus fruticosus* L., anthocyanins, stable food colorants

## Abstract

Given the importance of colour in the general acceptance or rejection of a product, the use of colorants is a widespread practice, particularly in the food industry. At the same time, with the increasing consumers’ awareness of the health effects that some artificial colorants can exert, there is a growing tendency to prioritize foodstuffs containing natural additives. In this work, *Morus nigra* L. and *Rubus fruticosus* L. fruit juices were characterized in terms of anthocyanins, organic acids, free sugars, and tocopherols, as also regarding their bioactive properties. Given their richness in anthocyanins, this study also aimed to prepare different solid colouring formulations by the spray-drying technique, using as stabilizers maltodextrin and arabic gum. Six free sugars and two organic acids were detected in the fruit juices, as well as the four tocopherol isoforms. Two cyanidin derivatives were found in *M. nigra* (cyanidin-3-*O*-glucoside and cyanidin-*O*-rhamnoside) and other four in *R. fruticosus* (cyanidin-*O*-hexoside, cyanidin-3-*O*-glucoside, cyanidin-*O*-pentoside, and cyanidin-3-*O*-dioxaloilglucoside). The developed colouring formulations revealed a good stability over time, in terms of anthocyanin concentration and colour parameters, and revealed to be safe for consumption, either concerning their low microbial load and lack of cytotoxicity. Thus, they represent a promising natural alternative to the massively used artificial colorants.

## 1. Introduction

One of the first perceptions of food is its colour, a characteristic that highly influences consumers’ choice, since it leads to the creation of an idea of the flavour, odour, and composition of the food product [1]. Therefore, food colorants are one of the most used additives, being applied to restore the original colour of a foodstuff when it is lost by some technological or storage process, to change, or even to enhance the original coloration of food [2]. With the recent food industry interest in gradually replacing the commonly used artificial colorants by natural counterparts, an increasing number of research studies have been focusing the exploitation of natural resources to meet this challenge. For being natural, non-toxic, and water-soluble, anthocyanins have been widely studied as alternatives to the mostly used artificial food colorants. Along with a great colouring capacity, these compounds have recognized bioactive properties, acting as antioxidants and helping in the prevention of cardiovascular and neurological diseases, cancer, and diabetes, among others [2,3], which make them even more suitable for food application.

As examples of rich anthocyanin sources, *Morus nigra* L. and *Rubus fruticosus* L. are small fruits that can be found in Asia, Europe, America, and Africa [3,4]. They are of high research interest due to their demonstrated different biological properties, such as anti-inflammatory, sedative, emollients, hypoglycaemic, cytotoxic, antiseptic, antifungal, antibacterial, and hepatoprotective activity [5,6,7]. In most mulberries, the major phenolic compounds reported are cyanidin-3-glucoside, quercetin-3-rutinoside, and kaempferol-3-rutinoside, however, the presence of quercetin-3-glucoside, cyanidin-3-rutinoside, pelargonidine-3-glucoside, and pelargonidin-3-rutinoside has also been reported [8,9]. These fruits are also rich in fatty acids, with a prevalence of linoleic acid, sugars, mainly fructose and glucose, and organic acids, mostly citric and malic acids [3,10,11,12]. On the other hand, *R. fruticosus* contains as major anthocyanins cyanidin-3-glucoside, cyanidin-3-arabinoside, and cyanidin-3-galactoside, but malvidin-3-glucoside, pelargonidin-3-glucoside, cyanidin-3-xyloside, cyanidin-3-rutinoside, and cyanidin-3-malonylglucoside are also present in smaller amounts. As phenolic acids, it mainly contains gallic acid, protocatechuic acid, *p*-hydroxybenzoic acid, caffeic acid, *p*-coumaric acid, and ellagic acid [13]. This fruit is considered highly nutritious, being composed of 85% of water, 10% of carbohydrates, minerals (Mg, Fe, K, and Ca), and vitamins (A, B, C, K, and E). It also contains fructose and glucose, flavonoids such as kaempferol and myricetin, and in immature fruits some carotenoids such as all-*trans*-lutein and all-*trans*-zeaxanthin can also be found [4,14].

Despite their multiple health benefits, some of these fruits are not used for consumption for not presenting the suitable size or properties to be included in the market, constituting a food industry residueom. The recovery of these bioresidues for added-value additives development could contribute to a circular bioeconomy, minimizing urban waste management issues and the scarcity of resources, mainly caused by the growing urban population and the linear economy. As such, in the present study, *M. nigra* and *R. fruticosus* fruits were used for the development of natural food colorants with stable colouring properties and respecting safety parameters, along three months of storage at different conditions. Once these formulations have the purpose of being added to foodstuff, the chemical composition and bioactive properties of both fruit extracts were also evaluated.

## 2. Results and Discussion

### 2.1. Chemical Composition

#### 2.1.1. Free Sugars

The results obtained for the free sugars composition of *Morus nigra* L. and *Rubus fruticosus* L. are presented in Table 1. Both fruits revealed the presence of fructose, glucose, sucrose, trehalose and raffinose. In *R. fruticosus* another sugar was also found, but its identification was not possible. *M. nigra* revealed the highest total sugars concentration, with 449 ± 2 mg/g extract. For both fruit extracts, the most abundant sugar was fructose, followed by glucose (respectively 248 ± 2 and 229.9 ± 0.3 mg/g extract in *M. nigra* and 201 ± 1 and 163.5 ± 0.1 mg/g extract in *R. fruticosus*). In addition to these two main sugars, both *M. nigra* and *R. fruticosus* extracts revealed traces of raffinose (5.085 ± 0.23 and 12.14 ± 0.61 mg/g of extract), trehalose (3.49 ± 0.184 and 5.275 ± 0.12 mg/g of extract), and sucrose (2.695 ± 0.077 and 3.7 ± 0.2 mg/g of extract).

It was not possible to establish a direct comparison of the results obtained herein with those of other authors, since in the present study, the fruit juices were assessed, and not the freeze-dried fruits. Nevertheless, in previous studies performed by Gundogdu et al., and Özgen et al. [3,15], glucose was found in a higher concentration than fructose in *M. nigra* fruits, although these were also the main sugars detected.

Regarding *R. fruticosus*, the results obtained with the juices are in agreement whit those reported by Milivojević et al. [14], with fructose being present in higher concentrations than glucose. Despite the scarcity of studies reporting the presence of raffinose and trehalose in fruits, some authors have reported their presence in berries. For instance, Aksic et al. [16] detected trehalose and raffinose in three blueberry cultivars (‘Bluecrop’, ‘Duke’, and ‘Nui’), while Palonen et al. [17] reported the presence of raffinose in ‘Festival’, ‘Titan’, and ‘Willamette’ raspberries, and Vara et al. [18] of both raffinose and trehalose in ‘Kwely’ raspberries.

#### 2.1.2. Organic Acids

Organic acids are compounds that exert a great influence on the organoleptic properties of fruits and can help preserve their nutritional value, being highly used in the food industry either as antioxidants, acidulants, or preservatives depending on their nature [19]. In this study, high amounts of malic acid were found, as also lower concentrations of oxalic acid (Table 1). These acids were found in higher quantity in *M. nigra* (146.9 ± 0.6 and 14.91 ± 0.09 mg/g of extract, respectively) than in *R. fruticosus* (101.9 ± 0.2 and 5.52 ± 0.02 mg/g of extract).

The results obtained for malic acid in *M. nigra* are consistent with those reported by Koyuncu [19] in a study evaluating different genotypes of blackberries, however, for oxalic acid, the maximum value reported was below the levels found in the present work, which can be related to the different extraction methods. On the other hand, Kafkas et al. [20] reported values between 0.6 ± 0.7 mg/g extract and 11.0 ± 2.7 mg/g extract of malic acid for different blackberries of the *Rubus* family, confirming the prevalence of this one against the other acids. Nevertheless, these authors did not find oxalic acid and detected ascorbic and citric acids. The differences observed can, once again, be related to the fact that in the referred study, metaphosphoric acid was used to perform the extraction, whereas herein the freeze-dried juice was directly used for analysis. It is also important to highlight that the chemical composition of these fruits are affected by different factors such as the place of cultivation, temperature, humidity, soil, collection method and time, storage method, sample treatment, among others [14].

#### 2.1.3. Tocopherols

Tocopherols are the most important natural fat-soluble antioxidants in the nutritional area. In *M. nigra*, the four isoforms were found (Table 1), with the prevalence of α-tocopherol (43 ± 2 mg/g of extract), followed by γ-tocopherol (12.5 ± 0.2 mg/g of extract); and in lower amounts, δ-tocoferol (5.5 ± 0.1 mg/g of extract) and β-tocopherol (1.27 ± 0.03 mg/g of extract). On the other hand, in *R. fruticosus*, only α-tocopherol was found, in a concentration of 6.1 ± 0.1 mg/g of extract. Comparing the two species, it is evident that *M. nigra* shows nearly seven times more quantity of α-tocopherol than *R. fruticosus*, which may justify the better results obtained with *M. nigra* in the TBARS antioxidant assay.

In a study performed by Wajs-bonikowska et al. [21], the α-, γ-, and δ-tocopherol isoforms were found in samples of *R. fruticosus* collected in Poland; a supercritical CO_2_ method and Soxhlet extraction using two solvents (hexane and ethanol) were compared for the extraction of tocopherols from this fruit pomace and, in all cases, lower amounts of α-tocopherol (0.72 ± 0.06, 0.70 ± 0.05, and 0.51 ± 0.08, respectively) were obtained, comparing to the present work, which is also possibly explained by the different extracts assessed and corroborates the importance of consuming these fruits juice.

#### 2.1.4. Anthocyanins

Anthocyanins are natural pigments which colour can vary from blue to red tonalities. These molecules have been increasingly applied in food industry, not only for their great colouring capacity, but also for conferring bioactive properties to the products in which they are included. The attempt to identify anthocyanins by UPLC-DAD-ESI/MS analysis in *M. nigra* and *R. fruticosus*, was based on retention times (Tr), maximum absorption wavelengths of the UV-Vis region (max), pseudomolecular ion ([M]^+^) and molecular ion fragmentation (MS^2^), being the identification performed by comparison with available standards and/or literature data.

**Figure 1:**
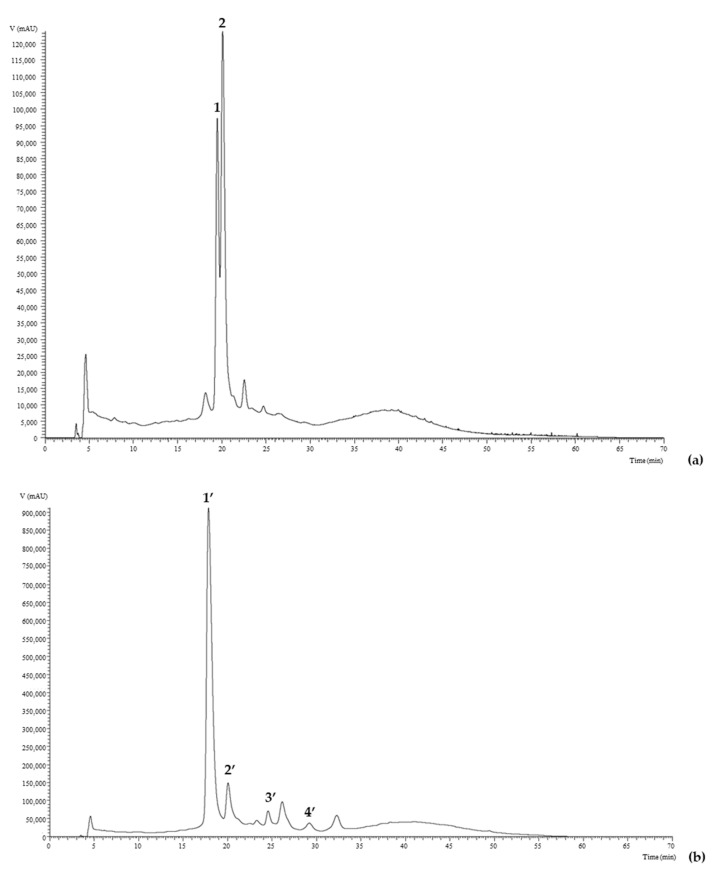
Chromatographic profile of the anthocyanin compounds found in *M. nigra* (**a**) and *R. fruticosus* (**b**) fruit juices, recorded at 520 nm (1: cyanidin-3-*O*-glucoside; 2: cyanidin-*O*-rhamnoside-*O*-hexoside; 1′: cyanidin-*O*-hexoside; 2′: cyanidin-3-*O*-glucoside; 3′: cyanidin-*O*-pentoside; 4′: cyanidin-3-*O*-dioxaloilglucoside).

In the *M. nigra* juice, two anthocyanins were identified (Table 2). Figure 1a presents the chromatographic profile of the anthocyanin compounds detected. Compound 1 (cyanidin-3-*O*-glucoside) was positively identified in comparison with the chromatographic and MS characteristics of the commercial standard. Compound 2 ([M]^+^ at *m*/*z* 595) presented two MS^2^ fragments, revealing two losses of a ramnose unit and a hexose unit (*m*/*z* at 287; −146 u and −162 u, respectively), therefore, it was identified as cyanidin-*O*-ramnoside-*O*-hexoside. These anthocyanins, especially the former, are very characteristic for being present in high quantities in this type of fruit, for example, Pawlowska et al. [8] recorded an amount of 17.9 mg/10 g of fresh Moraceae fruits.

The chromatographic analysis of *R. fruticosus* showed the presence of four anthocyanins (Table 2), all identified as cyanidin glycosidic derivatives. Figure 1b shows the chromatographic profile of anthocyanins present in this fruit. Compound 2′ (cyanidin-3-*O*-glucoside) was positively identified by comparison to the commercial standard. Compound 1′ ([M]^+^ at *m*/*z* 449) presented the same pseudomolecular ion as compound 2, with a loss of one hexose (−162 u). In this case, it was not possible to identify the position and nature of the hexose portion, because the peak retention times do not correspond to any of the available standards. Compound 3′ ([M]^+^ at *m*/*z* 419) lost −132 u, corresponding to a pentose, led to the compound tentative identification as cyanidin-*O*-pentoside. Compound 4′ ([M]^+^ at *m*/*z* 593) was identified as cyanidin-3-*O*-dioxaloilglucoside, considering the information described in literature, since this compound is found in different berries cultivars (marionberry_ORBC and blackberry) [22]. Cyanidin-3-*O*-glucose, a characteristic anthocyanin in blackberries, was found in both fruits, together with other cyanidin derivatives. The presence and variety of these anthocyanins is related to several health promoting properties, namely antioxidant, antitumor, anti-inflammatory, and antidiabetic, highlighting the importance of their consumption.

### 2.2. Bioactive Properties

*M. nigra* and *R. fruticosus* juices were used to formulate natural colorants for food application. As such, the extracts were processed using the spray-drying technique, through which three powder formulations were prepared: one only using the fruit juice, a second formulation using the juice and maltodextrin (40%), and a third one using 20% of a 1:1 (*w*/*w*) maltodextrin arabic gum mixture, as described in Section 3.4.2. The formulations, as well as the extracts, were assessed in terms of antioxidant, antibacterial, and antifungal properties, given the importance of these properties in foodstuff, not only for conferring health benefits, but also for delaying food oxidation and microbiological spoilage.

#### 2.2.1. Antioxidant Activity

The antioxidant activity of *M. nigra* and *R. fruticosus* extracts, as well as that of the different colouring formulations, was determined by two in vitro assays, the lipid peroxidation inhibition assays (TBARS) and the oxidative haemolysis inhibition assay (OxHLIA). 

In the case of *M. nigra* (Table 3), a high antioxidant activity of the extract was evidenced in TBARS assay with an IC_50_ value of 39 ± 2 µg/mL, which is almost four times lower than that of the positive control, Trolox (139 ± 5 µg/mL).

Likewise, all the colouring formulations presented a high antioxidant activity, very similar among them, which despite being higher than that presented by the extract, were significantly lower than that of Trolox. Regarding the OxHLIA assay, a concentration of 569 ± 14 µg/mL of the extract was able to delay the oxidative haemolysis for 120 min, which despite being a higher concentration than that needed for the positive control, can be considered a good antioxidant capacity for a natural extract. Furthermore, all the colouring formulations required a lower concentration than the extract, with the formulations containing maltodextrin and maltodextrin with arabic gum revealing the best antioxidant activity, with IC_50_ values of 286 ± 10 µg/mL and 296 ± 11 µg/mL, respectively. In a general perspective, the formulations prepared with maltodextrin and maltodextrin with arabic gum presented the best antioxidant activity in both assays without significant differences between them. The antioxidant potential of mulberry fruit was also assessed in previous studies; for example, Arfan et al. [23] reported the antioxidant potential of methanol and acetone sugar-free extracts, determined through ABTS, DPPH (2,2-diphenyl-1-picrylhydrazyl), and reducing power assays. On the other hand, Do et al. [24] reported the antioxidant capacity (DPPH) of spray-dried mulberry juice. Once in the present study, cellular-based assays were employed, it was not possible to compare the results.

In what concerns *R. fruticosus* (Table 4), for TBARS assay, the extract presented a great antioxidant activity, with an IC_50_ value of 100 ± 2 µg/mL, which is significantly lower than that obtained with the positive control, Trolox (139 ± 5 µg/mL). In fact, all the colouring formulations presented a better antioxidant activity than Trolox, being the control formulation the one that presented the highest activity (IC_50_ value of 78.4 µg/mL). In OxHLIA assay, the extract needed a higher concentration to delay the oxidative haemolysis for 120 min, 215 ± 3 µg/mL, compared to that needed for Trolox (183 ± 4 µg/mL). With respect to the colouring formulations, a similar behaviour was observed, comparing to TBARS, with the control formulation presenting the best antioxidant activity, in a concentration of 194 ± 6 µg/mL, a result close to that obtained for Trolox. On the other hand, the formulations prepared with maltodextrin and maltodextrin with arabic gum presented IC_50_ values of 250 ± 4 µg/mL and 248 ± 5 µg/mL, respectively, which are slightly higher than that obtained for the control, but still represent great results. The antioxidant activity of blackberry powders was also reported by Ferrari et al. [25], but the results are not directly comparable to the ones obtained herein given the different spray-drying conditions and antioxidant test applied (DPPH scavenging activity).

#### 2.2.2. Antimicrobial Activity

The results obtained for *M. nigra* (Table 3) and *R. fruticosus* (Table 4) extracts evidenced that both fruits present activity in all Gram-positive and Gram-negative bacteria assessed, showing very similar minimum inhibitory concentrations (MIC) and minimum bactericidal concentrations (MBC). All MIC and MBC values are higher than those showed by the positive controls, nevertheless, these are based on natural extracts, which despite having a lower activity, are not associated with negative effects for health, as some antibiotics. Among the Gram-positive bacteria, *M. nigra* and *R. fruticosus* revealed a better effect on *B. cereus*, both in terms of minimum concentrations necessary to inhibit the growth (5.01 and 5.03 mg/mL, respectively) and in the minimum concentrations necessary to have a bactericidal effect (10.02 and 10.06 mg/mL, respectively). *E. coli* was the bacterium belonging to the Gram-negative group in which better inhibitory activity was evidenced by both fruits, presenting the same MIC value (2.50 mg/mL). However, *M. nigra* presented a lower bactericidal capacity (MBC: 5.01 mg/mL) than *R. fruticosus* (MBC: 2.51 mg/mL). In a previous study performed by Četojević-Simin et al. [26], blackberry bagasse extracts also revealed the capacity to inhibit the growth of *E. coli*, *S. typhymurium*, *Pseudomonas aeruginosa*, *S. aureus*, *S. saprophyticus*, *B. cereus*, and *L. monocytogenes*, but the results are not directly comparable to the ones obtained herein, given the different extracts assessed and the applied method (disk diffusion method). Regarding mulberry, Khalid et al. [27] also reported the antibacterial activity of the fruit juice against *Bacillus spizizenii*, *Bacilus subtilis*, *Corynebacterium diphtheride*, *Enterococcus faecalis*, *S. aureus*, *E. coli*, *P. aeruginosa*, and *S. typhymurium*.

In terms of antifungal capacity, both *M. nigra* and *R. fruticosus* extracts evidenced inhibitory activity in all the tested fungi, although in higher concentrations than the positive controls. For all fungi, the extracts presented activity in similar concentrations, with the exception of *A. versicolor*, for which *M. nigra* presented a MIC of 2.51 mg/mL, which was much lower than that showed by *R. fruticosus* (MIC: 20.12 mg/mL). In addition, *T. viride* was the most sensitive fungus for both extracts (MIC: 1.25 mg/mL).

Regarding the colouring formulations of *M. nigra* (Table 3), despite presenting higher MIC and MBC values than the positive controls, streptomycin and ampicillin, all of them presented antibacterial properties, with the control formulation revealing activity in lower concentrations than the colorants containing maltodextrin and maltodextrin with arabic gum. Besides, these formulations also showed antifungal capacity against most of the assessed fungi. The colorant containing maltodextrin and arabic gum was the most effective, especially against *T. viride* and *A. versicolor*, in the same concentrations (MIC: 4.26 mg/mL; MFC: 8.52 mg/mL). However, none of the three formulations presented fungicidal activity for *P. ochrochloron*, nor the control formulation or the formulation with maltodextrin against *A. niger*.

Regarding the antibacterial activity of the formulations prepared with *R. fruticosus* (Table 4), it can be observed that although presenting higher inhibitory and bactericidal concentrations than the positive controls, they all present bioactivity, especially the control formulation against Gram-positive bacteria, except for *B. cereus*, which revealed a higher sensitivity for the colouring formulation with maltodextrin (MIC: 2.51 mg/mL; MBC: 5.02 mg/mL). For Gram-negative bacteria, better results were obtained with the colouring formulation containing maltodextrin with arabic gum, which, in most cases, showed effective concentrations twice lower than those obtained with maltodextrin and the control.

Generally, the bioactivity achieved with *R. fruticosus* colouring formulations was not very different, being the one prepared with maltodextrin the one presenting the best antifungal capacity, with the lowest inhibitory and fungicidal concentrations for *A. versicolor* (MIC: 1.26 mg/mL; MFC: 2.51 mg/mL), *A. niger* (MIC: 3.77 mg/mL; MFC: 5.02 mg/mL), and *P. ochrochloron* (MIC: 2.51 mg/mL; MFC: 5.02 mg/mL). However, the other two colorants presented similar results, with the control formulation exhibiting the best MIC and MFC values for *P. funiculosum* (1.27 and 2.53 mg/mL, respectively) and *T. viride* (0.91 and 1.27 mg/mL).

### 2.3. Stability of the Colouring Formulations

To assess the stability of the developed colorants, the anthocyanin concentration and colour parameters were assessed over 12 weeks of storage at refrigerated (3 °C) and room (23 °C) temperatures, to ensure their colouring capacity. Moreover, to guarantee their safety to be applied in food products, their microbial load and cytotoxicity for non-tumour cells were also evaluated. In terms of colour and anthocyanin concentration, the analyses were performed after preparation (t0) and after 4, 8, and 12 weeks of storage. In what concerns the safety assessment, the colouring formulations were analysed after preparation (t0) and after 12 weeks of storage.

#### 2.3.1. Colour and Anthocyanin Concentration

**Figure 2:**
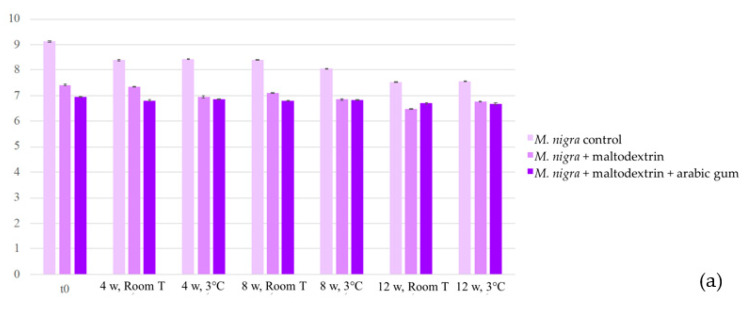

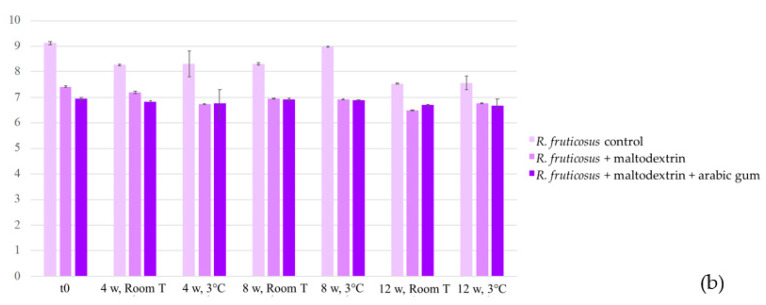
Evolution of anthocyanin concentration (mg/g) of the colouring formulations from *M. nigra* (**a**) and *R. fruticosus* (**b**). w: weeks, Room T: room temperature.

In the analysis performed along the three months of storage, the colouring formulations revealed the same anthocyanin qualitative composition as the extracts (results presented in Section 2.1.4), with *M. nigra* formulations presenting cyanidin-3-*O*-glucoside and cyanidin-*O*-rhamnoside, and *R.fruticosus* containing cyanidin-*O*-hexoside, cyanidin-3-*O*-glucoside, cyanidin-*O*-pentoside, and cyanidin-3-*O*-dioxaloylglucoside. Thus, the total anthocyanin concentration was considered to measure these compounds variation along the storage time, both at room and refrigerated temperatures (Figure 2). 

In previous studies performed by Ferrari et al. [25,28], where blackberry pulps were subjected to spray-drying, this technique also revealed to be effective on anthocyanins preservation. Similar conclusions were achieved with mulberry juice powders, in studies employing different spray-drying parameters [24].

**Figure 3:**
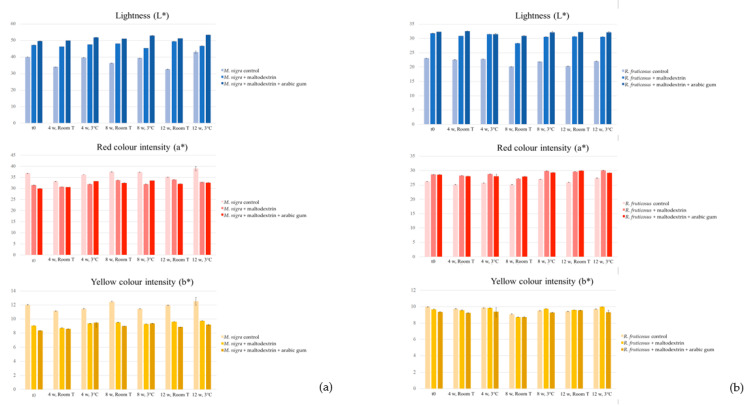
Evolution of colour parameters, L*, a*, and b* (CIE L*a*b* units) of the colouring formulations from *M. nigra* (**a**) and *R. fruticosus* (**b**). w: weeks, Room T: room temperature. In any case, no substantial differences were observed in any of the parameters analysed, either as a function of storage time (up to 12 weeks), or the temperature at which the samples were stored (23 °C or 3 °C).

In terms of colour assessment, the parameters luminosity (L*), green/red hue (a*) and yellow/blue hue (b*) were determined and compared (Figure 3).

Considering *R. fruticosus*, the formulations containing maltodextrin and a mixture of maltodextrin and arabic gum revealed higher values of luminosity and red colour hue, what was not observed in terms of yellow colour. Comparing these results with the ones obtained for the total anthocyanin content, despite the higher concentration in the control formulation (which was expected since the other formulations contain 40% adjuvants), a stronger red hue was verified in the formulations containing maltodextrin and maltodextrin + arabic gum.

Concerning the results obtained for *M. nigra* formulations, the higher luminosity measured in these samples is remarkable when compared to that observed in the formulations of *R. fruticosus*. On the other hand, the formulation prepared with maltodextrin + arabic gum was once again the one showing the best results (greater luminosity), although in this case, with more significant differences compared to the formulation prepared only with maltodextrin, which was much better than the control formulation. In terms of intensity of red and yellow hues, and although the values recorded were also higher than those measured in the homologous formulations of *R. fruticosus*, it was verified that the formulations prepared with adjuvants did not allow to obtain the same colour levels as the control formulation. Finally, the results of anthocyanin levels were practically identical to those quantified in the solid formulations of *R. fruticosus*.

#### 2.3.2. Microbial Analysis 

##### Evaluation of Pasteurization Efficiency

According to the Food and Drug Administration juices’ rule 66 FR 6137, these beverages must be developed assuring that a reduction of 5-log of the pathogenic microorganism is achieved [29].

From Table 5, it can be seen that the contaminated sample without thermal treatment was also able to reduce the number of microorganisms. This capacity exhibited by these samples can be explained by the colorant´s characteristics, namely the antimicrobial activity provided by these molecules. Nevertheless, it still not being efficient in controlling the growth of these contaminants. Also, it can be stated that the microorganisms were inoculated at 10^9^ cell/mL, but, immediately after the contamination procedure, in some cases they were found at 10^7^–10^8^. This can be due to the adjustment of these microorganisms in the densitometer that also consider dead cells, thus justifying the difference between the inoculated and the real counting’s.

Analysing the applied thermal treatments, it can be observed that at 80 °C, the microorganisms *E. coli* and *B. cereus* were apparently completely eliminated, while the yeast and the moulds still presented significative colony counts. On the other hand, when the temperature of 90 °C was applied, none of the microorganisms was detected, meaning that this temperature is the most efficient in the elimination of these microorganisms, allowing the preservation of the developed colorants. Although the samples treated with 80 °C revealed capacity to reduce more than 5-log of the tested microorganisms, the samples treated with 90 °C revealed the highest log reduction without compromising the integrity of the colouring compounds.

Therefore, the pasteurization at 90 °C was selected to prepare the samples for further analysis.

##### Microbial Load in the Final Juice Samples

The microbial load (log_10_ CFU/g) of the formulations was assessed to guarantee their safety for consumption (Table 6 and Table 7). The results were analysed through a two-way ANOVA, which allowed an individualized understanding of each factor. Table 6 and Table 7 represent the microbial load of the mesophilic aerobic microorganisms, coliforms, yeasts, and moulds of the *R. fruticosus* and *M. nigra* formulations, respectively. The two analysed times (after preparation and after 12 weeks) are represented in the upper part of the table for each storage temperature, while in the lower part, in each of the formulations, both times are included. This allows for a better understanding of the influence of each factor (Formulation or Storage Time) on the outcome.

Considering *R. fruticosus* samples stored at room temperature, Table 6 shows that, in addition to the fact that no coliforms were detected in any sample, at any of the times, there was a significant interaction between factors for yeasts and moulds, and some trends can be extracted from the estimated marginal means. It was also possible to verify that, for yeasts, the control registered the presence of these contaminants at t0, however, after three months they were not detected, while, for the samples with maltodextrin, they were not detected at any time. In terms of moulds, it was also clear that, with time, all samples tended to undetected values, although initially, the maltodextrin formulations registered a lower value than the control. Finally, it was possible to verify that the type of formulation was decisive in the case of the mesophilic aerobic microorganisms, since these were only recorded in the control samples, registering a significant difference between them. Regarding storage time, there were no significant differences between t0 and 12 weeks after preparation.

Regarding the samples stored at refrigerated temperature (Table 6), it is possible to verify that there was a significant interaction for both factors in all microorganisms. Unlike the samples stored at room temperature, in the refrigerated formulations a trend to increase over time was evidenced. Although in the samples with maltodextrin there were no values for these microorganisms at t0, in the control samples values between 3 and 4 log10 CFU/g were already evident. In the maltodextrin samples, yeasts and coliforms were not detected at any time of analysis. Comparatively, storage at room temperature seems to be more beneficial in the control of microorganisms, which presence decreased over time, especially for yeasts and moulds.

Regarding *M. nigra* colouring formulations stored at room temperature (Table 7), it was possible to verify once again that there was a significant interaction between storage time and formulation type, with all microorganisms (ST × F < 0.05). Again, coliforms were not detected in any sample; as for yeasts, no contamination was recorded in the formulation containing maltodextrin with Arabic gum, and in the case of moulds they were not detected in any of the samples containing maltodextrin.

**Figure 4:**
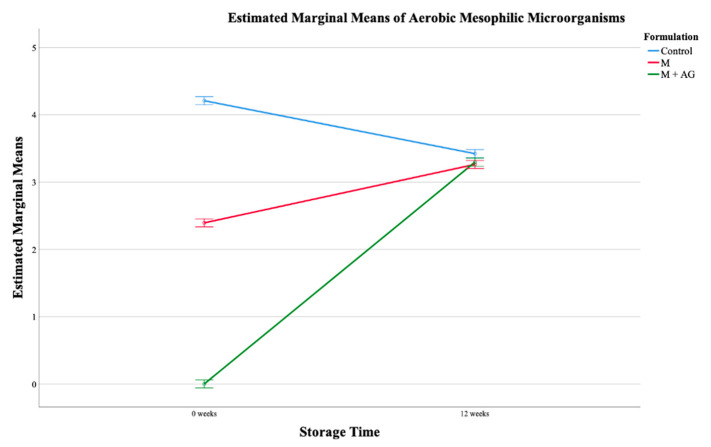
Estimated marginal mean plot of the aerobic mesophilic microorganisms for *M. nigra* at room temperature.

In what concerns the refrigerated *M. nigra* colorants, a significant interaction was recorded for all microorganisms, which revealed that both time and type of formulation contributed in an interactive manner to the behaviour of the samples, except for moulds, where the influencing parameter was the type of formulation, showing that the formulations containing adjuvants did not present or presented less contamination than the control formulation. From the estimated marginal means, it can be verified that, initially, the mesophilic aerobic microorganisms in the control had a higher count, while the samples that contained maltodextrin did not present these microorganisms, but after 12 weeks all samples showed the same load of these microorganisms (Figure 4).

However, after 12 weeks of storage, the control decreased the values of these microorganisms, while both samples with maltodextrin increased. It should be noted that coliforms were not detected in any sample; and in the samples containing maltodextrin and arabic gum, yeasts and moulds were not detected. In addition, regarding moulds, the only sample that had this contaminant in t0 maintained the same microbial load during the 12 weeks, while in the samples with maltodextrin it increased between storage times.

#### 2.3.3. Cytotoxicity

In terms of cytotoxicity, none of the formulations revealed activity against a primary culture of porcine liver cells, which indicates that they are safe for incorporation in foodstuff without presenting any risk for consumers’ health.

## 3. Materials and Methods

### 3.1. Samples

**Figure 5:**
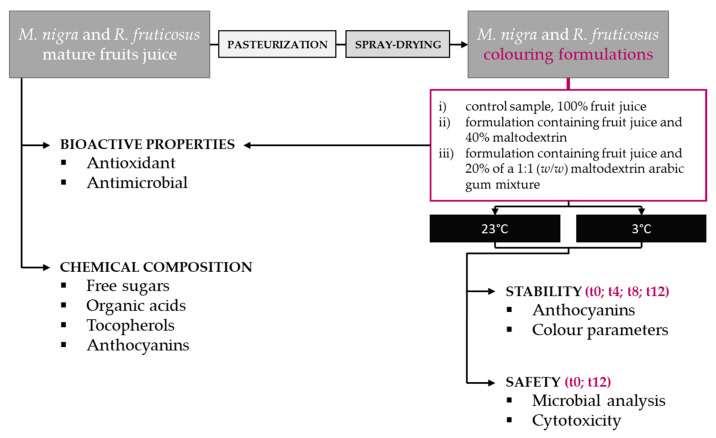
Overview of the analyses performed with the fruits juice and colouring formulations.

Mature fruits of *Rubus fruticosus* L. and *Morus nigra* L. were collected in Trás-os-Montes, Portugal, and provided by “Ponto Agrícola Unipessoal, Lda.” (Baião, Portugal), respectively. They were blended to obtain a juice rich in anthocyanins and the juice was centrifuged, filtered through Whatman No. 4 filter paper, frozen, and lyophilized for further analysis and preparation of the colouring formulations. The juice was reconstituted by dissolving in water to a final concentration of 100 mg/mL. Figure 5 provides an overview of the analyses performed.

### 3.2. Chemical Composition

#### 3.2.1. Free Sugars

For free sugars assessment, the lyophilized extract was dissolved in water to a final concentration of 100 mg/mL. The analysis was performed according to a procedure previously described by the authors, by HPLC (Knauer, Smartline system 1000, Berlin, Germany) coupled to a refractive index detector (RI detector, Knauer Smartline 2300) [30]. A Eurospher 100-5 NH2 column (250 mm × 4.6 mm, 5 µm, Knauer) was used to achieve the separation at 35 °C, through isocratic elution with acetonitrile/deionized water (70:30, *v*/*v*) at a flow rate of 1 mL/min. The results were expressed in mg per g of extract.

#### 3.2.2. Organic Acids

The lyophilized extract was dissolved in water in a concentration of 100 mg/mL and the analysis was performed by ultra-fast liquid chromatography (UFLC) coupled to a photodiode array detector (PDA), through a methodology described by Pereira et al. [31], using a Shimadzu 20A series UFLC (Shimadzu Corporation, Kyoto, Japan). The compounds were separated using a SphereClone reverse phase C18 column (250 mm × 4.6 mm, 5 µm, Phenomenex, Torrance, CA, USA), at 35 °C and a flow rate of 0.8 mL/min of sulphuric acid (3.6 mM), through isocratic elution. The results were expressed in mg per g of extract.

#### 3.2.3. Tocopherols

To assess the tocopherols composition, the lyophilized extract was subjected to an extraction procedure, according to the conditions described by Barros et al. [30]. Briefly, the extract was added with BHT and IS (tocol) solutions, and the mixture was homogenized with ethanol. Hexane and NaCl were added, vortex-mixing after each addition, and the mixture was centrifuged to recover the upper layer. The extract was, then, dried under a nitrogen stream, redissolved in hexane, and filtered through a 0.22 µm disposable LC filter disk. The analysis was achieved by HPLC (Knauer Smartline system 1000) using a FP-2020 fluorescence detector (Jasco, Japan), using a Polyamide II normal-phase column (250 mm × 4.6 mm, 5 µm, YMC Waters, Milford, MA, USA) for compounds separation, with isocratic elution with n-hexane and ethyl acetate (70:30, *v*/*v*), at 35 °C and a flow rate of 1 mL/min. The quantification was performed through the internal standard method and the results were expressed in mg per g of extract.

#### 3.2.4. Anthocyanins

For anthocyanins analysis, the extract or colouring formulation was dissolved in water to a final concentration of 5 mg/mL, filtered (0.2 µm), and injected in an HPLC equipment (Dionex Ultimate 3000 UPLC, Thermo Scientific, San Jose, CA, USA) coupled to a diode-array detector (280, 330, 370, and 520 nm wavelengths) and an electrospray ionization mass spectrometer (Linear Ion Trap LTQ XL, Thermo Scientific) working in positive mode, as previously described by Gonçalves et al. [32]. For compounds separation, an AQUA^®^ reverse phase C_18_ column (5 μm, 150 mm × 4.6 mm, Phenomenex) was used, at 35 °C, using previously described gradients [32]. The anthocyanins determination was performed according to their retention time, UV-Vis and mass spectra, in comparison with authentic standards and using literature data. The quantification was achieved using a seven levels calibration curve obtained for different standard compounds. The results were expressed in mg per g of extract/formulation.

### 3.3. Bioactive Properties

#### 3.3.1. Antioxidant Properties

Two methods were applied to assess the antioxidant activity of the extracts and colouring formulations. Through the TBARS (thiobarbituric acid reactive substances) assay with porcine (*Sus scrofa*) brain homogenates, the decrease in TBARS formation was measured to assess the lipid peroxidation inhibition capacity, according to Pereira et al. [33]. The oxidative haemolysis inhibition assay (OxHLIA) was performed with sheep erythrocytes to evaluate the anti-haemolytic activity for 60 and 120 min, following a previously described procedure [34]. In both assays, the results were expressed in µg/mL (EC_50_ values; extract/formulation concentration required to obtain 50% of antioxidant activity) and Trolox was used as positive control.

#### 3.3.2. Antimicrobial Properties

For the antimicrobial analysis, six fungi (*Aspergillus fumigatus* (ATCC 1022), *Aspergillus versicolor* (ATCC 11,730), *Aspergillus niger* (ATCC 6275), *Penicillium funiculosum* (ATCC 36,839), *Penicillium ochrochloron* (ATCC 9112), and *Trichoderma viride* (IAM 5061)), three Gram-positive (*Bacillus cereus* (food isolate), *Staphylococcus aureus* (ATCC 6538), and *Listeria monocytogenes* (NCTC 7973)), and three Gram-negative (*Escherichia coli* (ATCC 35,210), *Enterobacter cloacae* (human isolate)*,* and *Salmonella* Typhimurium (ATCC 13,311)) bacteria were used. The antifungal and antimicrobial activities were assessed according to Soković et al. [35,36] and the results were expressed as minimum inhibitory (MIC), minimum bactericidal (MBC), and minimum fungicidal (MFC) concentrations, in mg/mL. As positive controls, ketoconazole and bifonazole were used for fungi and streptomycin and ampicillin for the bacterial strains.

### 3.4. Colouring Formulations

#### 3.4.1. Pasteurization

The lyophilized extract was dissolved in water to a final concentration of 100 mg/mL and was submitted to a pasteurization procedure, previously described by Molina et al. [37]. Briefly, four hermetic bags containing the dissolved extract were maintained in a water bath at 90 °C for 60 s and were subsequently cooled in ice until achieving 3 °C. One of these bags was used to evaluate the microbial load, as described in Section 3.5.2., and the remaining samples were stored at 3 °C for ~15 h for further spray-drying.

#### 3.4.2. Spray-Drying

Three formulations were prepared by spray-drying: (i) a control sample, consisting of 100% fruit juice; (ii) a formulation containing fruit juice added with 40% maltodextrin; and (iii) a formulation composed of fruit juice added with 20% of a 1:1 (*w*/*w*) maltodextrin arabic gum mixture. The referred percentages were selected after testing different contents of maltodextrin and maltodextrin + arabic gum (1:1, *w*/*w*), and are expressed as the ratio of the drying adjuvants weight relative to the total solids weight in the prepared juice (100 mg/mL). These formulations were spray-dried according to the procedure of Moser et al. [38], analysing the efficiency of the drying process and the suitability of the used drying coadjutants, maltodextrin and arabic gum. The solutions were prepared immediately before atomization by mixing the fruit juice with the selected materials under stirring at room temperature for 10 min. A Mini Spray Dryer B-290 (Büchi, Flawil, Switzerland) programmed in the normal operation mode (nozzle diameter: 0.7 mm; atomized volume: 200 mL, solids content < 33%) was used. The optimized operation conditions were an inlet temperature of 140 °C, an outlet temperature of 72 °C, an aspiration of 90%, with the pump working at 20% (6 mL/min). To calculate the yield of the process, expressed in percentage, the ratio between the obtained powder weight and the initial solution’s total solids weight (dry-basis), was considered.

### 3.5. Stability of the Colouring Formulations

To assess the stability of the prepared formulations, after the spray-drying process, a portion of each powdered sample was separated to perform the required analyses immediately after preparation (t0) and the remaining quantity was divided into 2 equal portions to be stored at room (23 °C) and refrigerated (3 °C) temperatures, in sterile flasks protected from light. All the samples were analysed in order to evaluate the variation of their anthocyanin concentration and colour parameters after 4, 8, and 12 weeks of storage (comparing with t0), as well as their safety in what concerns the microbial load and cytotoxic properties at t0 and after 12 weeks of storage.

#### 3.5.1. Anthocyanin Concentration and Colour Parameters

To evaluate the concentration of anthocyanins in the different formulations, stored at distinct temperatures, these were dissolved in water to a final concentration of 5 mg/mL and were analysed as described in Section 3.2.4.

In what concerns the colour parameters, a colourimeter (model CR-400, Konica Minolta Sensing, Inc., Osaka, Japan) equipped with a specific tool for granular materials (model CR-A50) was used to analyse the powders, as reported by the authors Pereira et al. [39]. For that purpose, the colour parameters were obtained in the CIE L*a*b* colour space, through the illuminant C with a diaphragm aperture of 8 mm, and the Spectra Magic Nx software (version CM-S100W 2.03.0006, Konica Minolta) was used to analyse the obtained data.

#### 3.5.2. Microbial Analysis

##### 3.5.2.1. Determination of the Pasteurization Procedure

The ideal pasteurization conditions were established after a microbial contamination procedure that allowed to choose the optimal conditions capable of reducing “pertinent microorganisms” in at least 5-log cycles, as determined by the Food and Drug Administration rule 66 FR 6137 for fruit juices [29]. For this, juice samples (10 mL, in triplicate) were contaminated with a mix of *Escherichia coli, Bacillus cereus, Aspergillus parasiticus, *and* Zygosaccharomyces rouxii* (provided by the Mountain Research Centre of the Polytechnic Institute of Bragança, Bragança, Portugal), with approximately 10^9^ cell/mL of each microorganism. The contamination process was performed as described by Fernandes et al. [40]. The samples were then submitted to two pasteurization procedures: 90 °C for 60 s and 80 °C for 60 s (following the procedure described in Section 3.4). The contaminated samples were analysed for microbial load immediately before and after pasteurization. The viable cells were further assessed according to the described in Section 3.5.2.2.

##### 3.5.2.2. Microbial Load of the Final Colouring Formulations Subjected to the Chosen Pasteurization Conditions

The microbiological analysis was assessed by mixing 1 g of the powdered samples with 9 mL of peptone water. These solutions were diluted until achieving 10−6 and the following counts were performed, as previously described by Molina et al. [37]: aerobic plate count (total viable count; ISO 4833-2:2013), coliforms (and *Escherichia coli*; ISO 4832:2006), yeasts and moulds (ISO 21527-1/2:2008), and *Bacillus cereus* (ISO 7932:2004).

#### 3.5.3. Cytotoxicity

The cytotoxicity in non-tumour cells was performed by monitoring the cells growth using a phase contrast microscope. Briefly, a primary culture of porcine liver non-tumour cells was sub-cultured and plated in 96 well plates (density of 1.0 × 10^4^ cells/well) with the culture medium Dulbecco’s modified Eagle’s medium (DMEM) containing FBS (10%), penicillin (100 U/mL), and streptomycin (100 μg/mL), according to the protocol described by Abreu et al. [41]. The results were expressed in μg/mL (GI_50_ values; formulation concentration required to inhibit 50% of the net cell growth) and ellipticine was used as positive control. 

### 3.6. Statistical Analysis

Three samples were assessed for each analysis, in assays carried out in triplicate and the results were presented as mean values and standard deviation (SD). The results were treated using Student’s *t*-test at a 5% significance level or one-way analysis of variance (ANOVA) post-hoc Tukey. A Two-way ANOVA was used for the assessment of the stability of the formulations. The analyses were performed using the SPSS v.22.0 program (IBM SPSS version 22.0, IBM Corp., Armonk, NY, USA) using a significance of 0.05.

## 4. Conclusions

*Morus nigra* L. and *Rubus fruticosus* L. fruit juices were assessed for their chemical composition, namely in what concerns free sugars, organic acids, tocopherols, and anthocyanins. The samples revealed six sugars, two organic acids, and the four isoforms of tocopherols. In *M. nigra*, two anthocyanins were detected, cyanidin-3-*O*-glucoside and cyanidin-*O*-rhamnoside, whereas in *R. fruticosus*, four distinct cyanidin derivatives were identified, namely cyanidin-*O*-hexoside, cyanidin-3-*O*-glucoside, cyanidin-*O*-pentoside, and cyanidin-3-*O*-dioxaloilglucoside. The fruit juices were also analysed in terms of antioxidant and antimicrobial properties, revealing a strong bioactivity. Moreover, given their richness in anthocyanin compounds, the fruit juices were also used to prepare solid colorants for application in food industry. Three formulations were obtained through the spray-drying technique for each fruit, and their stability was assessed over 12 weeks of storage at room and refrigerated temperatures. In general, the colorants revealed a great and stable colouring capacity over time, without toxicity for non-tumour cells and microbial loads within the values acceptable for food. Thus, these fruits can be considered as good natural sources of anthocyanins to be used as natural colorants, not only in food industry, but also in pharmaceuticals, cosmetics, or textiles, among others.

## Figures and Tables

**Table 1:** Free sugars, organic acids, and tocopherols composition of *M. nigra* and *R. fruticosus* fruit extracts.

	*M. nigra*	*R. fruticosus*	*p*-Value
Free Sugars (mg/g extract)			
Fructose	248 ± 2	201 ± 1	<0.001
Glucose	229.9 ± 0.3	163.5 ± 0.1	<0.001
Sucrose	2.70 ± 0.08	3.7 ± 0.2	<0.001
Trehalose	3.5 ± 0.1	5.3 ± 0.1	<0.001
Raffinose	5.1 ± 0.2	12.1 ± 0.6	<0.001
Unknown	nd	21 ± 1	-
Total	449 ± 2	373 ± 1	<0.001
Organic acids (mg/g extract)			
Oxalic acid	14.91 ± 0.09	5.52 ± 0.02	<0.001
Malic acid	146.9 ± 0.6	101.9 ± 0.2	<0.001
Total	161.8 ± 0.6	107.3 ± 0.2	<0.001
Tocopherols (mg/g extract)			
α-Tocopherol	43 ± 2	6.1 ± 0.1	<0.001
β-Tocopherol	1.27 ± 0.03	nd	-
γ-Tocopherol	12.5 ± 0.2	nd	-
δ-Tocopherol	5.5 ± 0.1	nd	-
Total	62 ± 2	6.1 ± 0.1	<0.001

nd: not detected; *p*-values obtained by applying the Student’s t-test at a 5% significance level.

**Table 2:** Anthocyanin composition of *M. nigra* and *R. fruticosus* fruit extracts.

Peak	Rt (min)	λ_max_ (nm)	[M]^+^ *m*/*z*	MS^2^	Tentative Identification	Concentration (mg/g Extract)
*M. nigra*						
1	19.72	515	449	287(100)	Cyanidin-3-*O*-glucoside	6.096 ± 0.003
2	22.00	517	595	449(31), 287(100)	Cyanidin-*O*-rhamnoside-*O*-hexoside	2.443 ± 0.002
					Total	8.538 ± 0.005
*R. fruticosus*						
1′	16.69	518	449	287(100)	Cyanidin-*O*-hexoside	3.761 ± 0.007
2′	19.55	518	449	287(100)	Cyanidin-3-*O*-glucoside	1.81 ± 0.01
3′	24.01	517	419	287(100)	Cyanidin-*O*-pentoside	1.265 ± 0.001
4′	30.14	519	593	287(100)	Cyanidin-3-*O*-dioxaloilglucoside	1.198 ± 0.001
					Total	8.03 ± 0.02

Calibration curve used for quantification: cyanidin-3-*O*-glucoside (*y* = 134,578*x* – 3 × 10^6^; *R^2^*: 0.9986; LOD: 0.25 µg/mL; LOQ: 0.83 µg/mL).

**Table 3:** Bioactive properties of *M. nigra* fruit extract and colouring formulations.

	Antioxidant Activity (IC_50_ Values, µg/mL)						
		*M. nigra* extract	*M. nigra* control	*M. nigra* + M	*M. nigra* + M + AG	Trolox ^1^	
TBARS assay		39 ± 2a	55.6 ± 0.4c	51 ± 1b	52 ± 2b	139 ± 5	
OxHLIA assay	60 min	253 ± 10d	166 ± 5c	124 ± 5b	108 ± 5a	85 ± 2	
	120 min	569 ± 14d	324 ± 17c	286 ± 10a	296 ± 11a	183 ± 4	
	**Antibacterial activity (MIC and MBC values, mg/mL)**						
		*M. nigra* extract	*M. nigra* control	*M. nigra* + M	*M. nigra* + M + AG	Streptomycin ^1^	Ampicilin ^1^
*Bacillus cereus*	MIC/MBC	5.01/10.02	6.81/6.81	8.52/8.52	8.52/8.52	0.10/0.20	0.25/0.40
*Staphylococcus aureus*	MIC/MBC	20.04/20.04	3.41/6.81	4.26/8.52	4.26/8.52	0.17/0.25	0.34/0.37
*Listeria monocytogenes*	MIC/MBC	10.02/20.04	3.41/3.41	4.26/4.26	4.26/4.26	0.20/0.30	0.40/0.50
*Escherichia coli*	MIC/MBC	2.50/5.01	3.41/3.41	4.26/8.52	4.26/8.52	0.20/0.30	0.40/0.50
*Enterobacter cloacae*	MIC/MBC	10.02/20.04	1.7/1.7	2.13/2.13	4.26/4.26	0.043/0.25	0.086/0.37
*Salmonella* Typhimurium	MIC/MBC	10.02/20.04	1.7/3.41	2.13/4.26	4.26/4.26	0.20/0.30	0.75/1.20
	**Antifungal activity (MIC and MFC values, mg/mL)**						
		*M. nigra* extract	*M. nigra* control	*M. nigra* + M	*M. nigra* + M + AG	Ketoconazole ^1^	Bifonazole ^1^
*Aspergillus fumigatus*	MIC/MFC	5.01/10.02	13.63/27.27	17.05/17.05	8.52/17.05	0.38/0.95	0.48/0.64
*Aspergillus versicolor*	MIC/MFC	2.51/5.01	6.81/27.27	4.26/8.52	4.26/8.52	0.20/0.50	0.10/0.20
*Aspergillus niger*	MIC/MFC	20.04/>20.04	27.27/>27.27	34.09/>34.09	17.05/34.09	0.20/0.50	0.15/0.20
*Penicillium funiculosum*	MIC/MFC	2.51/5.01	13.63/27.27	17.05/34.09	8.52/34.09	0.20/0.50	0.20/0.25
*Penicillium ochrochloron*	MIC/MFC	2.51/5.01	27.27/>27.27	34.09/>34.09	34.09/>34.09	1.00/1.50	0.20/0.25
*Trichoderma viride*	MIC/MFC	1.25/2.51	2.13/13.63	2.13/4.26	4.26/8.52	1.00/1.00	0.15/0.20

^1^ Positive controls; *M. nigra* control: colouring formulation control; *M. nigra* + M: colouring formulation containing maltodextrin (40%); *M. nigra* + M + AG: colouring formulation containing maltodextrin (20%) and arabic gum (20%). IC_50_: extract concentration providing 50% of antioxidant activity; MIC: minimum inhibitory properties; MBC: minimum bactericidal concentration; MFC: minimum fungicidal properties. For the antioxidant activity, different letters in each line mean significant differences (*p* < 0.05).

**Table 4:** Bioactive properties of *R. fruticosus* fruit extract and colouring formulations.

	Antioxidant Activity (IC_50_ Values, µg/mL)						
		*R. fruticosus* extract	*R. fruticosus* control	*R. fruticosus* + M	*R. fruticosus* + M + AG	Trolox ^1^	
TBARS assay		100 ± 2c	78.4 ± 0.8a	101 ± 2c	94.9 ± 0.2b	139 ± 5b	
OxHLIA assay	60 min	120 ± 7c	81 ± 3a	108 ± 5b	106 ± 5b	85 ± 2a	
	120 min	215 ± 3b	194 ± 6a	250 ± 4c	248 ± 5c	183 ± 4a	
	**Antibacterial activity (MIC and MBC values, mg/mL)**						
		*R. fruticosus* extract	*R. fruticosus* control	*R. fruticosus* + M	*R. fruticosus* + M + AG	Streptomycin ^1^	Ampicilin ^1^
*Bacillus cereus*	MIC/MBC	5.03/10.06	2.53/5.06	2.51/5.02	5.03/10.06	0.10/0.20	0.25/0.40
*Staphylococcus aureus*	MIC/MBC	13.41/20.12	5.06/13.49	20.08/20.08	10.06/20.12	0.17/0.25	0.34/0.37
*Listeria monocytogenes*	MIC/MBC	10.06/20.12	5.06/10.12	10.04/20.08	10.06/26.83	0.20/0.30	0.40/0.50
*Escherichia coli*	MIC/MBC	2.51/2.51	1.27/2.53	2.51/5.02	1.26/2.52	0.20/0.30	0.40/0.50
*Enterobacter cloacae*	MIC/MBC	13.41/20.12	5.06/10.12	10.04/20.08	5.03/10.06	0.043/0.25	0.086/0.37
*Salmonella typhimurium*	MIC/MBC	13.41/20.12	5.06/10.12	10.04/20.08	5.03/10.06	0.20/0.30	0.75/1.20
	**Antifungal activity (MIC and MFC values, mg/mL)**						
		*R. fruticosus* extract	*R. fruticosus* control	*R. fruticosus* + M	*R. fruticosus* + M + AG	Ketoconazole ^1^	Bifonazole ^1^
*Aspergillus fumigatus*	MIC/MFC	5.03/10.06	2.53/5.06	2.51/5.02	5.03/10.06	0.38/0.95	0.48/0.64
*Aspergillus versicolor*	MIC/MFC	20.12/>20.12	1.27/2.53	1.26/2.52	1.26/2.52	0.20/0.50	0.10/0.20
*Aspergillus niger*	MIC/MFC	20.12/>20.12	5.06/10.12	3.77/5.02	5.03/10.06	0.20/0.50	0.15/0.20
*Penicillium funiculosum*	MIC/MFC	2.52/5.03	1.27/2.53	2.51/5.02	2.52/5.03	0.20/0.50	0.20/0.25
*Penicillium ochrochloron*	MIC/MFC	2.52/5.03	5.06/10.12	2.51/5.02	5.03/10.06	1.00/1.50	0.20/0.25
*Trichoderma viride*	MIC/MFC	1.26/2.52	0.91/1.27	2.51/5.02	1.26/2.52	1.00/1.00	0.15/0.20

^1^ Positive controls; *R. fruticosus* control: colouring formulation control; *R. fruticosus* + M: colouring formulation containing maltodextrin (40%); *R. fruticosus* + M + AG: colouring formulation containing maltodextrin (20%) and arabic gum (20%). IC_50_: extract concentration providing 50% of antioxidant activity; MIC: minimum inhibitory properties; MBC: minimum bactericidal concentration; MFC: minimum fungicidal properties. For the antioxidant activity, different letters in each line mean significant differences (*p* < 0.05).

**Table 5:** Microbial counts of the analysed samples inoculated with approximately 10^9^ cells/mL of each tested microorganism and submitted to pasteurization (*n* = 3).

	Initial Counts (before Pasteurization) (log_10_ CFU/mL)	Counts after Pasteurization at 80 °C (log_10_ CFU/mL)	Counts after Pasteurization at 90 °C (log_10_ CFU/mL)	log_10_ Cycle Reduction in Contaminated Samples without Pasteurization	log_10_ Cycle Reduction after Pasteurization at 80 °C	log_10_ Cycle Reduction after Pasteurization at 90 °C
*E. coli*	5.36 ± 0.02	nd	nd	3.57	8.93	8.93
*B. cereus*	3.46 ± 0.06	nd	nd	3.75	7.21	7.21
*A. parasiticus*	4.44 ± 0.05b	2.82 ± 0.05a	nd	4.33	5.96	8.77
*Z. rouxii*	3.59 ± 0.03b	2.31 ± 0.08a	nd	4.02	5.26	7.61

nd—not detected; different letters in each line mean significant differences (*p* < 0.05).

**Table 6:** Microbial analysis (log10 CFU/g) of the colouring formulations of *R. fruticosus* stored at room and refrigerated temperature for 12 weeks.

		Aerobic Mesophilic Microorganisms	Coliforms	Yeasts	Moulds
Room temperature					
Storage time (ST)	0 weeks	1 ± 2	nd	1 ± 2	3.14 ± 0.19
	12 weeks	1 ± 2	nd	nd	nd
*p-*value (*n* = 15)	Student’s *t* test	0.407	-	<0.001	<0.001
Formulation (F)	Control	3.5 ± 0.1	nd	2 ± 2	2 ± 2
	M	nd	nd	nd	1 ± 2
	M + AG	nd	nd	nd	2 ± 2
*p-*value (*n* = 10)	Tukey’s HSD test	<0.001	-	<0.001	<0.001
ST×F (*n* = 30)	*p-*value	0.498	-	<0.001	<0.001
Refrigerated temperature					
Storage time (ST)	0 weeks	1 ± 2	nd	1 ± 1	3.1 ± 0.2
	12 weeks	3.4 ± 0.7	nd	1 ± 1	2.9 ± 0.1
*p-*value (*n* = 15)	Student’s T test	<0.001	<0.001	<0.001	<0.001
Formulation (F)	Control	3.8 ± 0.2	nd	2 ± 2	3.2 ± 0.2
	M	2 ± 2	nd	nd	2.89 ± 0.08
	M + AG	1 ± 1	nd	1 ± 2	3.15 ± 0.06
*p-*value (*n* = 10)	Tukey’s HSD test	<0.001	-	<0.001	<0.001
ST × F (*n* = 30)	*p-*value	<0.001	-	<0.001	<0.001

nd—not detected. In each row, for storage time, represents a statistically significant difference between the two time-points of analysis, while for the formulation, different letters mean a statistically significant difference, with a significance of 0.05. The standard deviations presented were calculated using the results obtained in different conditions, and as such, they should not be considered as precision measures, but as a range of values.

**Table 7:** Microbial analysis (log10 CFU/g) of the colouring formulations of *M. nigra* stored at room and refrigerated temperature for 12 weeks.

		Aerobic Mesophilic Microorganisms	Coliforms	Yeasts	Moulds
Room temperature					
Storage time (ST)	0 weeks	2 ± 2	nd	2 ± 1	1 ± 1
	12 weeks	3.0 ± 0.4	nd	2 ± 1	1 ± 1
*p-*value (*n* = 15)	Student’s T test	<0.001	-	<0.001	0.032
Formulation (F)	Control	3.7 ± 0.5	nd	3.12 ± 0.06	2.9 ± 0.1
	M	2.5 ± 0.1	nd	3.0 ± 0.3	nd
	M + AG	2 ± 2	nd	nd	nd
*p-*value (*n* = 10)	Tukey’s HSD test	<0.001	-	<0.001	-
ST × F (*n* = 30)	*p-*value	<0.001	-	<0.001	0.016
Refrigerated temperature					
Storage time (ST)	0 weeks	2 ± 2	nd	2 ± 1	1 ± 1
	12 weeks	3.32 ± 0.08	nd	2 ± 1	2 ± 2
*p-*value (*n* = 15)	Student’s T test	0.028	-	0.114	0.942
Formulation (F)	Control	3.8 ± 0.4	nd	3.05 ± 0.09	2.9 ± 0.2a
	M	2.8 ± 0.5	nd	3.0 ± 0.3	2 ± 2b
	M + AG	2 ± 2	nd	nd	nd
*p-*value (*n* = 10)	Tukey’s HSD test	<0.001	-	<0.001	<0.001
ST × F (*n* = 30)	*p-*value	<0.001	-	<0.001	0.855

nd—not detected. In each row, for storage time, represents a statistically significant difference between the two time-points of analysis, while for the formulation, different letters mean a statistically significant difference, with a significance of 0.05. The standard deviations presented were calculated using the results obtained in different conditions, and as such, they should not be considered as precision measures, but as a range of values.

## Data Availability

The data presented in this study are available in this article.
